# The investigation of diverse physiological and therapeutic impact of cellular-based products derived from human cumulus cells

**DOI:** 10.55730/1300-0152.2626

**Published:** 2022-09-19

**Authors:** Derya BURUKÇU, Sema YILMAZ, Oya ALAGÖZ, Cumhur Kaan YALTIRIK, Erkut ATTAR, Fikrettin ŞAHİN, Esra AYDEMİR

**Affiliations:** 1 Department of Genetics and Bioengineering, Faculty of Engineering, Yeditepe University, İstanbul, Turkey; 2 Department of Internal Medical Sciences and - Child Health and Diseases, Uşak University, Uşak, Turkey; 3 Department of in vitro Diagnostic, Yeditepe University Medical School and Yeditepe University Hospital, İstanbul, Turkey; 4 Department of Neurosurgery, Ümraniye Training and Research Hospital, İstanbul, Turkey; 5 Department of Neurosurgery, Yeditepe University Medical School and Yeditepe University Hospital, İstanbul, Turkey; 6 Department of Biomedical Engineering, Faculty of Engineering and Natural Sciences, Biruni University, İstanbul, Turkey

**Keywords:** Cumulus cells, hyaluronic acid, differentiation, nucleus pulposus cells

## Abstract

Particular somatic cells, namely cumulus cells (CCs) that support the oocyte maturation, fertility, and viability by providing the nutrients and energy to the oocyte envelop the mammalian oocyte. In this study, discarded human cumulus tissues were used to reveal the value of hyaluronic acid-rich CCs on several cellular events, including differentiation. Conditioned media, recovered from the primary culture of CCs, were introduced to the human nucleus pulposus cells (hNPCs) which were functionally distorted because of the loss of chondrogenecity. Enlightening the impact of cumulus conditioned media (CCM) on wound healing and angiogenesis was also investigated. In line with these goals, differentiation of hNPCs into chondrocytes with CCM as the basal medium containing traditional differentiation agents was induced upon isolation and characterization of hCCs and hNPCs. The effects were detected by differentiation-specific cell stains and gene expression analyses. Scratch and tube formation assays were performed to detect the effect of CCM on wound healing and angiogenesis. Our results showed that cumulus cell-conditioned media promoted the chondrogenesis and osteogenesis of hNPCs. A significant increase in angiogenesis and ability for wound closure was detected only in groups cultured in CCM compared to the control. These findings demonstrated that CCM might be used in therapeutics.

## 1. Introduction

**Figure 1:**
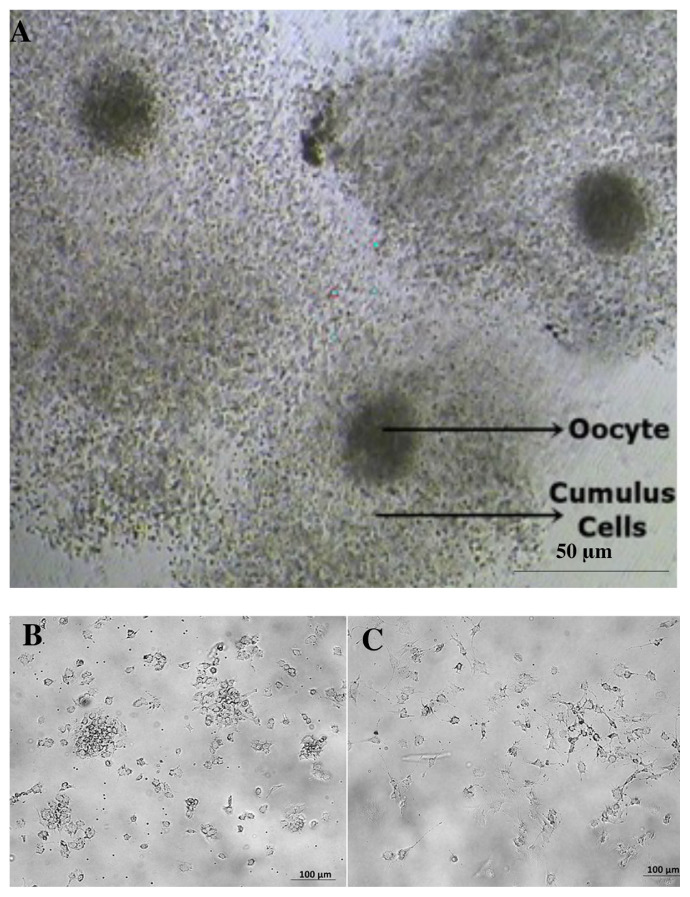
Microscopic images of CCs. The image taken at the IVF laboratory shows the CCs surrounding the oocytes (A), scale bar: 50 μM. hCCs at early days (1–3 days) (B), late days (4–7 days) (C), scale bar: 100 μm.

Cumulus cells (CCs) directly surround the oocyte and create a suitable environment for the development of a healthy oocyte (Dumesic et al., 2015). The tightly compact structure formed by the combination of the CCs and the oocyte is called a cumulus-oocyte complex (COC) (Figure 1), which comprises 3 to 4 concentric layers of CCs that can hold up to 2000 cells (Dale and Elder 2020). COC becomes clear in antral follicles and is surrounded by follicular fluid. Aspiration of this fluid, required for oocyte collection, is a standard step of in vitro fertilization (IVF) procedure. CCs have a very special extracellular matrix (ECM) structure that is rich in hyaluronic acid (HA), which is one glycosaminoglycan (GAGs) and responsible for “cumulus expansion” or “mucification” that takes a role in ovulation, fertilization, or determination of the oocyte quality. The studies investigating the characteristics of human CCs were very limited and used in the studies involved in reproduction (Chermuła et al., 2020).

Mesenchymal stem cells (MSCs) are one of the most attractive types of stem cells as they have a differentiation capacity, manipulation flexibility, and require relatively simple isolation procedures (Dominici et al., 2006). The studies prove the effect of HA in improved osteogenic and chondrogenic differentiation capacity of MSCs (sulfated HA) that inspires the scientists for designing biomaterials for the regeneration of tissues, including bone and cartilage. For example, in the cases of bone defects, commercially available HA scaffolds enriched in transforming growth factor-beta (TGFβ) protein stimulated the bone marrow-derived-MSCs to differentiate into chondrogenic lineages and generated cartilage-like structures in vitro in addition to the increased expression levels of chondrogenic markers. In a different study involved in osteogenic differentiation of MSCs, HA-based hydrogels improved the cell adhesion without the aid of osteogenic medium (Williams et al., 2003; Pfeifer et al., 2016). A knockout mouse model generated by the deficiency of the Has2, a well-known gene encoding the hyaluronan synthase -a marker for cumulus cells-has failed to develop the spine and stated the importance of HA (Roughley et al., 2011).

The spine structure, which is composed of vertebra defining bone segments, ligaments, and discs, is connected by the intervertebral disc (IVD) (Kayalioglu 2009), which makes the spine flexible without losing an abundant amount of strength and helps the movement of the body. The core component of IVD is the nucleus pulposus (NP) wrapped around by collagen-rich annulus fibrosus (AF). NP is made up of mostly water, sulfated-GAGs, type II collagen, and chondrocyte-like cells, which render the NP flexible against stress conditions. The tear or breaking open annulus fibrosus structure leads the NP to escape from its boundaries, which is defined as “disc herniation”. NP cells can be isolated for developing regenerative approaches by unique solutions or methods to remove only the herniated disc part. This study aimed to explain the potential of cellular products derived from human cumulus cells on restoring the cartilaginous property of hNPCs along with the investigation of the role of cumulus cell derivatives on stem cell differentiation, wound healing processes, and angiogenesis.

## 2. Materials and methods

### 2.1. Specimen collection

Tissue specimens were collected as follows: Human cumulus cells (hCCs) were collected from women who underwent IVF treatment (ages between 20 and 40) and a hernia tissue from a 36-year-old male patient who underwent surgery for lumbar disc herniation. All specimens were provided according to the ethical standards of the institutional review board of Yeditepe University Hospital (KAEK No:1281).

### 2.2. Primary cell cultures

Overall, to restore the cartilaginous properties, firstly, the primary hNPCs were generated, characterized, and further treated with cumulus cell-conditioned media consisting of chondrogenic differentiation agents. Both tissue types were isolated through mechanical and enzymatic digestion methods (Lee et al., 2007). Briefly, the nucleus pulposi tissues were divided into small pieces with a scalpel, digested with 3% pronase enzyme (#10165921001, Roche, USA) in serum-free DMEM (DMEM-LG, #12320032, Thermo Fisher Scientific, USA), followed by collagenase I (07415, Stemcell Technologies, Canada) diluted serum-free medium and incubated overnight at 37 °C. The cells at passage 5 were characterized by gene expression and surface marker analyses.

Cumulus tissues were cleaned from the excessive blood residues by washing with DPBS repeatedly and further digested by harsh pipetting in RPMI 1640 media (#21875034, Thermo Fisher Scientific, USA) containing 10% (v/v) heat-inactivated fetal bovine serum (FBS, #16140071, Thermo Fisher Scientific, USA) and 1% (v/v) Penicillin-Streptomycin-Amphotericin B (PSA, #15240062, Thermo Fisher Scientific, USA). Cells were transferred to collagen type I coated T25 flasks for overnight incubation. On alternate days, the media of hCCs were collected for up to a week, filtered with 0.22-μm filters (#FJ13ASCCA002DL01, GVS Filter Technologies, UK), and kept at −80 °C until use. After this point, the collected media will be referred to as cumulus-conditioned medium (CCM). Cells at passage 0 were simultaneously collected for characterization by cumulus specific gene expression analysis.

As for morphological analyses, once the cells (hCCs and hNPCs) adhered to the flask surfaces and acquired a stable morphology, images were captured under an inverted microscope (Carl Zeiss Microscopy, Germany) with various objective lenses (4X and 10X). The morphology of the cells was compared with the ones in the literature.

### 2.3. Characterization of stem cell markers of hNPCs by flow cytometer

To determine the stem cell characteristics of hNPCs, specific surface proteins were detected by flow cytometry as described in our previous study (Aydemir et al., 2012). Cell pellets were harvested and resuspended with 2% paraformaldehyde (PFA) for the fixation at +4 °C for 30 min. The conjugated antibodies were purchased from the Abcam (UK) as listed; CD90 (#ab95700, Abcam, Cambridge, MA), CD73 (#ab157335), CD34 (#ab18227), CD14 (#ab82434), CD105 (#ab53321), CD31 (#ab27333), CD44 (#ab58754), CD29 (#ab27314), CD45 (#ab134202), and CD117 (#ab130410). The samples were incubated overnight with various antibodies at +4 °C in dark. The samples were then analyzed in BD FACSCalibur™ (Becton Dickinson, USA).

### 2.4. Gene expression analyses

Human cumulus cells were initially characterized for their specific cellular markers including angiogenic genes. In summary, RNAs were isolated and reverse transcribed to complementary DNA (cDNA), amplified by conventional PCR, and confirmed by gel electrophoresis. Briefly, RNAs were isolated by combining two different methods namely Trizol lysis (#BS410A, Bio Basic, Canada) and silica column purification by NucleoSpin^®^ RNA kit (#740955.50, Macherey-Nagel, Germany). Complementary DNAs (cDNAs) were synthesized by QuantiTect Reverse Transcription Kit (#205313, Qiagen, Netherland) as described in the manufacturer’s instructions and used as templates for the quantification of various markers shown in Table 1. Conventional PCR reaction was performed with FastMix Frenche PCR Kit (#25401, iNtRON Biotechnology, Korea) according to the manufacturer’s instructions at MyCycler Thermal Cycler (#580BR, Bio-Rad, USA) with an annealing temperature of 60 °C.

HNPCs were also characterized by gene expression using Taqman PCR master mix (#A6121, Promega, USA) and performed by a conventional PCR method according to the manufacturer’s protocol. The chondrogenic markers were purchased from Thermo Fisher Scientific and depicted as follows; GAPDH (ID: Hs02786624_g1), Vimentin (ID:Hs00958111_m1), SRY-Box Transcription Factor 9 (SOX9) (ID: Hs01001343_g1), Aggrecan (ACAN) (ID: Hs00153936_m1), Forkhead Box F1 (FOXF1) (ID: Hs00230962_m1), Carbonic Anhydrase 12 (CA12) (ID: Hs01080909_m1), CD44 (ID: Hs01075864_m1), Cytokeratin 19 (CK19) (ID: Hs00761767_s1), and as for a housekeeping Beta-Actin (ACTB) (ID: Hs01060665_g1). Detection of cell specific genes were visualized under UV light (Chemidoc, Biorad, USA).

Chondrogenic and osteogenic differentiations were evaluated by quantifying the expressions of genes including collagen type II alpha-1 gene (COL2A1) and osteopontin (OPN), respectively, by a real-time PCR (SyBr Green) method with annealing/extension temperature of 60 °C and the gene expressions were normalized to 18S for the fold change analyses. For the fold change analysis, the 2^−ΔΔCT^ method was used as a relative quantification strategy for the data analysis (Rao et al., 2013). The primers for the markers are depicted in Table 1.

### 2.5. Cell viability assay for determining the nontoxic dose of CCM

The highest nontoxic dose of CCM to be given to the hNPCs was determined by 3-(4,5-Dimethylthiazol-2-yl)-5-(3-carboxymethoxyphenyl)-2-(4-sulfophenyl)-2H-tetrazolium (MTS), (#G1111, Promega, USA) assay. Shortly, hNPCs were seeded onto the four 96-well plates (#CLS6509, Corning, USA) at a density of 5 × 10^3^/well and treated with CCM diluted in regular culture medium of hNPCs (DMEM-LG containing 10% FBS and 1% PSA) at a 1:1 ratio for one time and measured for viability for four consecutive days (one plate/day). As for the untreated group, the sole culturing medium was used. At each day, one plate including the treated and untreated cells was treated with 10% MTS reagent prepared in 0.45% glucose in DPBS solution (#G8644, Sigma Aldrich, Germany) and incubated in a humidified chamber (5% CO_2_, 80% RH, and 37 °C) for 1 h in dark. Absorbance values of end-products of the reaction were determined by a plate reader (ELx800, Biotek Instruments, USA) at wavelength 490 nm. Lastly, the results were analyzed by comparing CCM diluted at a 1:1 ratio with the untreated control group.

### 2.6. Hyaluronic acid (HA) quantification in CCM

The amount of HA in the CCM was determined by the Hyaluronan Assay Kit (#AMS.CSR-HA-96KIT, Amsbio, USA) according to the manufacturer’s protocol. The measurement principle of the ELISA-like assay for HA is based on the competition method. Namely, a sample containing HA and Biotin-HABP are added to an HA-coated plate. Following addition of HRP-Avidin, HA binding Biotin-HAPB is detected by chromophoric substrate. In our method, briefly, wells were incubated with HA solution for coating for 1 h and then blocked with a blocking buffer for 30 min. Standards (provided in the kit), samples including various dilutions of CCMs, and RPMI-1640 containing 10% FBS as negative control were added to the treated wells as duplicates. Biotin-Hyaluronic acid-binding protein (HABP) was added to the wells and incubated for 1 h and followed by an HRP-Avidin introduction for another hour. The wells were thoroughly washed, applied with a substrate solution and incubated for 30 min in dark. The stop solution was added to each well and the absorbances were measured at 450 nm wavelength with a plate reader. The calibration curve was plotted against the absorbances of standards and the concentrations of HA in CCMs were determined.

### 2.7. Differentiation assays

Cells were resuspended in chondrogenic medium which was described in a previous study (Ciuffreda et al., 2016), diluted with CCM in a 1:1 ratio, and seeded as a droplet with a density of 4000 cells/μL.

As for the experimental groups; cells which were treated with low-glucose media consisting 10% FBS and labeled as negative control cells, treated with high-glucose media and labeled as positive control cells and finally treated with a mixture of high glucose media and cumulus conditioned media (1:1 ratio) and labeled as experimental group cells. Cells were grown in the culture for 21 days to complete the differentiation with refreshing media three times a week. The differentiation medium was prepared freshly every week. Upon completion, the images for morphological changes in hNPCs were taken under the inverted microscope (Carl Zeiss, Germany). Spreading of the cells from the droplets were captured and compared among the groups.

### 2.8. Detection of differentiation by alcian blue and alizarin red staining

After 2 weeks of differentiation, the images of morphological changes in hNPCs were captured under the inverted microscope. Osteoblast structures formed at differentiated groups were observed and compared among the groups. To detect the calcium deposits, the cells were stained with alizarin red staining (ARS). Briefly, the cells were washed with DPBS gently, fixed with absolute ethanol (#920.026, Isolab, Germany), and allowed to dry completely. ARS solution (#CM-0058, Lifeline Cell Technologies, USA) was introduced to the wells and was incubated for 15 min at room temperature (RT). The wells were rinsed with dH_2_O three times, treated with a leaching solution that contained 20% (v/v) methanol (#947.043, Isolab, Germany) and 10% (v/v) acetic acid for 15 min to extract the ARS stain. The absorbance of the samples was measured at 450 nm by a plate reader.

Chondrogenic differentiation was measured by the alcian blue staining. Shortly, cells were fixed with 4% PFA for 30 min at +4 °C, washed with DPBS, and stained with the solution for 30 min at RT in dark. Lastly, the images of the wells were captured, and the average blue color intensity was analyzed by the ImageJ image processing program developed at the National Institutes of Health (NIH, USA).

### 2.9. Wound healing assay

The effect of CCM on wound closure was quantified as described in our previous study (Coban et al., 2017). Shortly, human immortalized keratinocytes (HaCat, #300493, DKFZ, Heidelberg) were seeded onto six-well plates at 95% confluency. The following day, the medium was discarded and the wells were washed with DPBS (for the removal of floating cells) very gently ensuring not to remove the attached cells. By using a sterile tip, a scratch was made in the center of the well and cells were incubated with CCM. Images were taken every 6 h for 12 h. The effects on wound closure was determined and evaluated with Wimasis image analysis program (Wimasis, Spain).

### 2.10. Tube formation assay

The tube formation assay was performed to determine the effect of CCM and hCCs on vascularization. The effect of these groups was compared with human umbilical vein endothelial cells (HUVECs, #CRL-1730, ATCC, USA), a well-known in vitro model for the tube formation assay. Briefly, prechilled 48-well plates were coated with Matrigel (#354248, Corning, USA), and incubated at 37 °C for polymerization. HUVECs and hCCs were seeded onto matrigel-coated wells at a density of 70,000 cells/well as duplicate. The cells were treated with two different media and incubated at humidified chamber for 5–7 h and images of each group were captured by using an inverted microscope. Analysis of branch numbers in obtained images was done by an image analysis program, Wimasis.

### 2.11. Statistical analysis

Experiments were carried out in triplicates to enable a proper statistical analysis. GraphPad Prism 7.0 program was used for multiple comparisons among the experimental and control groups. One-way analysis of variance (ANOVA) with Tukey’s post hoc test was applied to data to determine the significance of differences. The *p < 0.05 was chosen as statistically significant.

## 3. Results

### 3.1. Morphology of hCCs

HCCs were isolated from the tissue surrounding the oocytes collected for in vitro fertilization processes mechanical digestion. Oocytes with cumulus cells’ layers before the removal of cumulus tissue which is required for spot-on intracytoplasmic sperm injection were demonstrated. The oocytes were located in the center and the cumulus cells formed a puffy layer around those oocytes (Figure 1A). HCCs were monitored under an inverted microscope for a week and the morphology of the cells was saved. The images of the cells in the early days of culture and the days before the harvest were captured as shown below. In the early days, hCCs were small and rounded (Figure 1B) but with their transition they gained a different look such as spindle-shaped with growing arms, probably creating junctions as time progresses. At the end of the week, the senescent period of hCCs began. During this period, the spindles disappeared, the cells became larger and their morphology was distorted (Figure 1C).

Gene expressions specific to cumulus cells along with angiogenic markers were verified to ensure whether desired cells were generated. Genes specific to hCCs including angiogenic markers were amplified and analyzed with gel electrophoresis and are depicted in Table 2. Overall, different samples expressed the genes specific to cumulus cells; therefore, a mixture of cumulus conditioned media (CCM) from each sample was prepared for further analyses.

### 3.2. Characterization of hNPCs by morphology, gene expression, and stem cells marker profiles

HNPCs were isolated through the enzymatic methods. The characterization of the cells was provided by the morphological analysis as well as gene expression profiling. In addition to these, mesenchymal stem cell properties of the cells were also detected by flow cytometry as listed in Table 3. Flow cytometry analysis showed that the cells possessed mesenchymal surface markers while not the hematopoietic ones (Figure S1).

**Figure 2:**
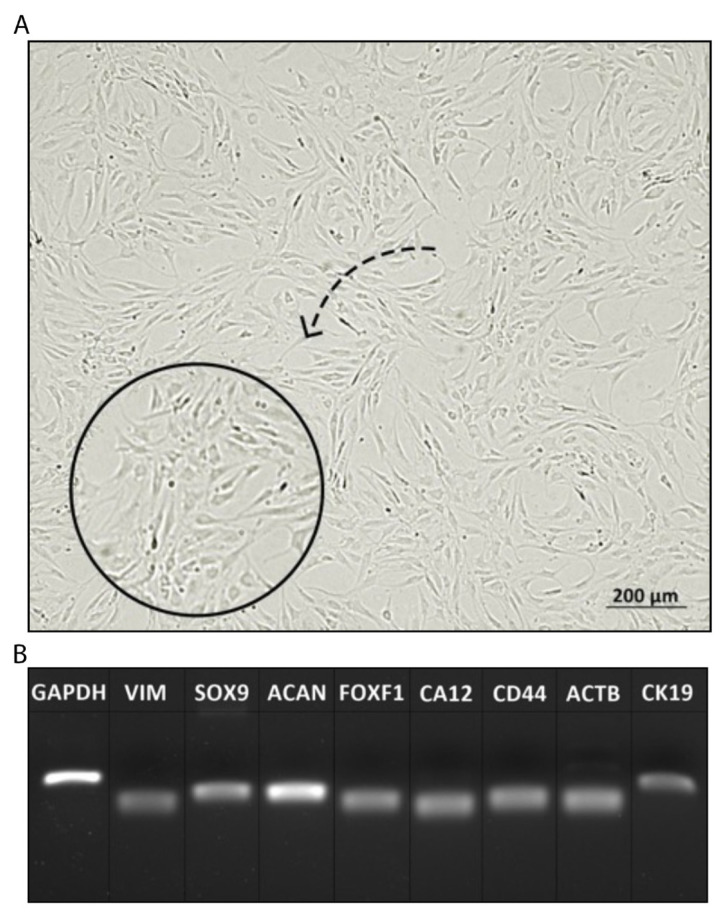
Microscopic images of hNPCs (A), scale bar: 200 μm. Gene expressions specific to hNPCs were illustrated by gel electrophoresis (B).

Upon the isolation, hNPCs were cultured in a sustained period and their morphologies were examined under the inverted microscope regularly. The images of the cells were displayed (Figure 2A). According to morphologic observations, hNPCs were giant and fusiform-shaped and had a homogenous structure. Genes specific to hNPCs, chondrification, and other mesenchymal markers including SOX9, AGGRECAN, CD44, VIMENTIN, FOXF1, CK12, and CA12 were analyzed by PCR detection. Gene quantification was visualized under UV light (Figure 2B) and according to it, the genes were found to be expressed by the hNPCs. While cells expressed Aggrecan in a higher amount, other genes seemed to be expressed less. However, all the genes that were expressed affirmed the characteristics of hNPCs.

### 3.3. Determination of nontoxic CCM concentration

The toxicity of CCM dilution at a 1:1 ratio on hNPCs was investigated by MTS assay (Figure S2). It was observed that there was no significant difference in viability between the CCM (1:1) treated and untreated control groups of hNPCs for 4 days. CCM (1:1) dilution did not cause any toxic effect on hNPCs. Based on this result, a 1:1 dilution ratio was used in the rest of the assays.

### 3.4. Increased chondrogenicity in CCM

**Figure 3:**
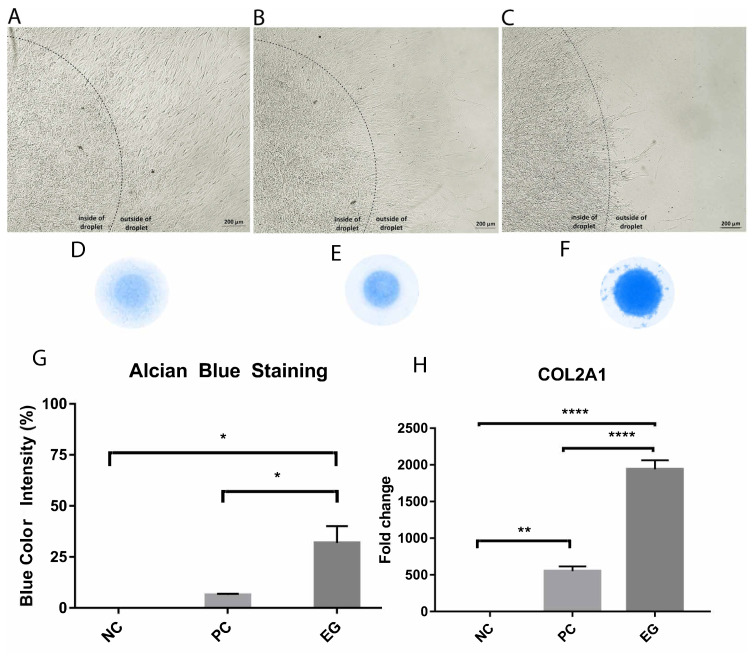
Chondrogenic differentiation analyses. Morphological changes upon chondrogenic differentiation of hNPCs on the 21st day. Negative control (A), positive control (B), and experimental group (C). The intensity of alcian blue is found to be the lowest in negative control (D), moderate in positive control (E), and highest in the experimental group (F).

Upon stimulation of chondrogenesis, conformational tests including alcian blue staining, and detection of COL2A1 expression were performed. A morphological comparison between differentiated and undifferentiated cells was also monitored. Results showed that negative control cells failed to maintain inside the limits of the initial droplet form. Cell proliferation continued around the droplet and the cells managed to leave out of the droplet (Figures 3A and 3B). However, the positive control cells (Figure 3C**)** stayed inside the droplet. The growth of the cells slowed down and the spread to the outside of the droplet decreased. The experimental group cells almost completely stopped falling out of the droplet.

Alcian blue staining was performed to detect the presence of sulfated glycosaminoglycans, the indicators for chondrogenesis at its higher level. The lowest intensity of the blue color was detected in the negative control cells (Figure 3D), a moderate intensity in the positive control cells (Figure 3E) whereas the most intensity was observed in the experimental group cells (Figure 3F). The intensity of blue color was quantified by ImageJ. Quantitative results were consistent with the observations (Figure 3G). The intensity of blue corresponded to 6% in the positive control, while it reached up to 32% in the experimental group. COL2A1, the main protein found in cartilage structures, was examined as a marker of chondrogenesis by Q-PCR. According to the PC cells, a 3-fold difference was detected in the experimental group (Figure 3H).

### 3.5. Enhanced osteogenicity by the induction of CCM

**Figure 4:**
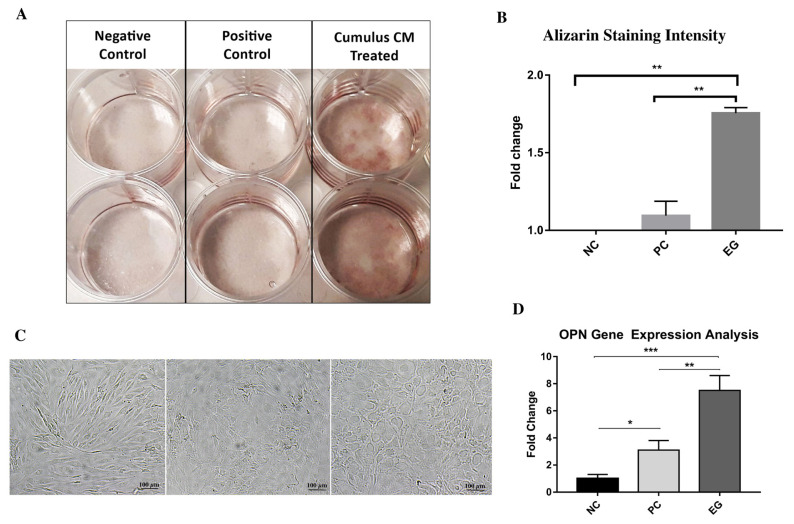
Osteogenic differentiation analyses. ARS was found to be the highest in CCM-treated cells (A). Graphical analysis shows the intensity of the ARS among the groups (B), morphological changes upon osteogenic differentiation on the 14th day (C). The expression levels of OPN was compared among the groups and found to be highly upregulated in the experimental group (D).

Both qualitative and quantitative tests for the determination of the osteogenic differentiation showed that the increased calcification was highest in the experimental group cells (Figure 4A). The quantitative analysis also confirmed the observations (Figure 4B). Absorbance of the ARS staining in the positive control was found to be 1.5-fold more than the experimental group. Morphology of the cells was also observed and the comparison between the groups was made. According to the observations, the untreated cells had fusiform shapes, whereas the differentiated cells gained more osteocyte-like round shapes. The number of rounded cells was higher in the experimental group (Figure 4C). Moreover, a significant increase was observed in the expression of the *OSTEOPONTIN (OPN*) gene. The EG cells showed 2-fold higher expression than the positive control cells and 4-fold higher than the negative control cells (Figure 4D).

### 3.6. Accelerated rate of wound closure by CMM

**Figure 5:**
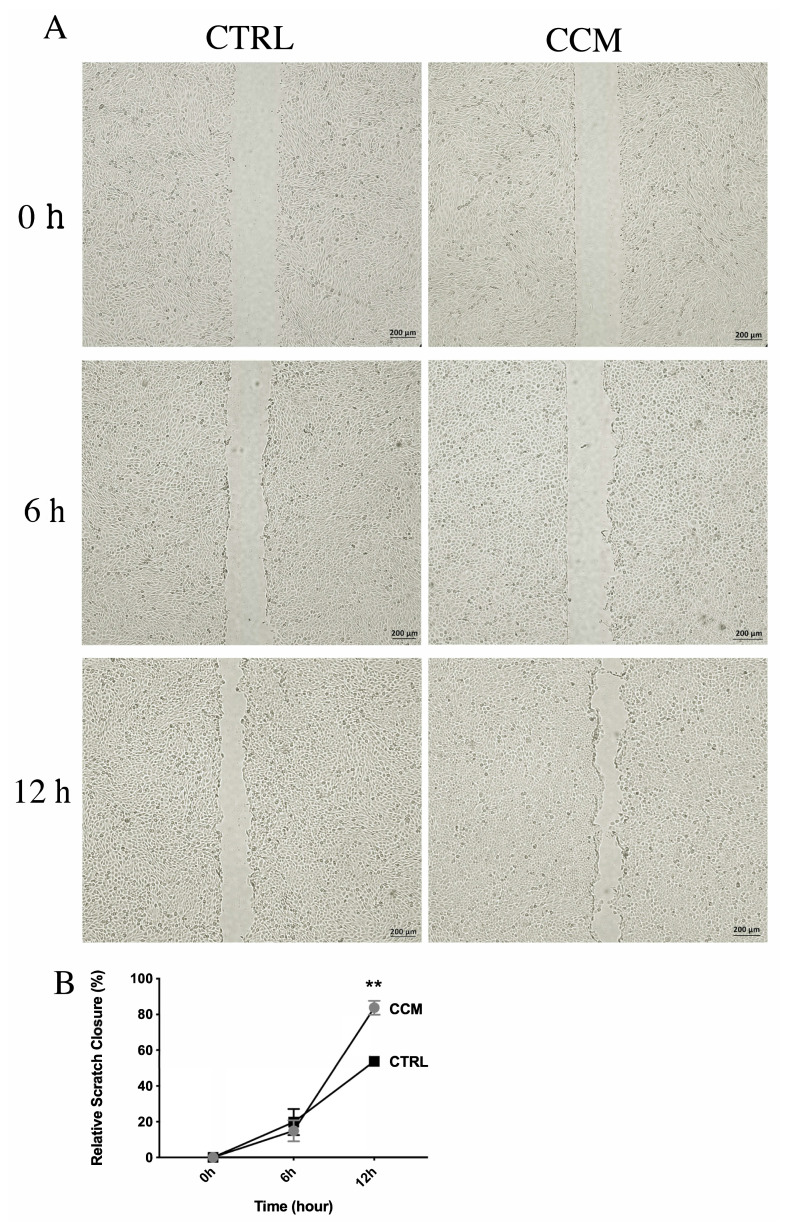
Wound closure assay. Images of closure area at different time points (0, 6, and 12 h) by CTRL cells and CCM-treated cells (A), scale bar: 200 μm. Analysis of scratch closure (B) (* p < 0.05).

According to the wound closure assay, there were no significant differences in the 6th h of treatment among the groups (Figure 5A). However, in the 12th h, the wound closure in the untreated control HaCat cells was 54%, whereas the CCM-treated HaCat cells closed the 84% of the gap (Figure 5B).

### 3.7. CCs as an alternative model for tube formation assay

**Figure 6:**
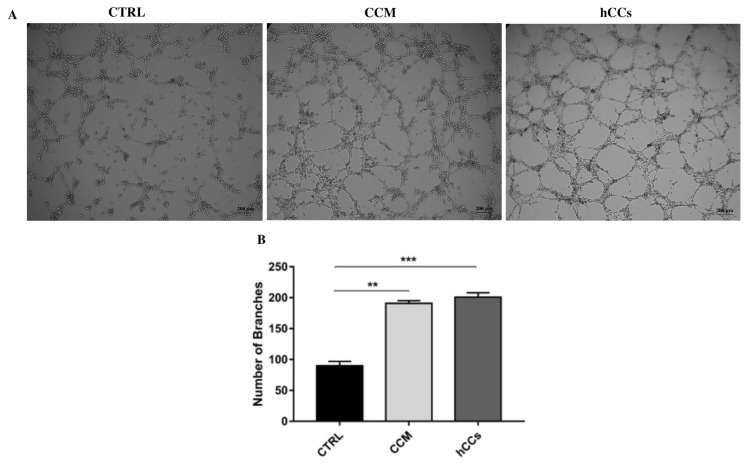
Tube formation analysis. Tube formation analyses showed the number of branches as microscopic images, from left to right: CTRL (HUVECs, CCM-treated HUVECs, and hCCs alone) (A), scale bar: 200 μm. Analysis was made by Wimasis (B) (* p < 0.05).

The effect of CCM and hCCs on angiogenesis was investigated. It was shown that there was a significant difference in the numbers of branches among groups. Control group cells (HUVECs) formed the least number of branches, whereas CCM-treated HUVEC cells had a higher number of branches. On the other hand, hCCs alone formed the highest number of branches approximately by 2 when compared to the others (Figure 6A). The numbers of branches were also analyzed by Wimasis and illustrated graphically (Figure 6B).

### 3.8. CCM consists of the significant amount of hyaluronic acid

Hyaluronic acid amount in CCM was measured by an ELISA-like assay. According to the results, CCM consisted of a high amount of HA; 102.8 ng/mL (Figure S3). Compared to the positive group (RPMI with FBS), it was seen that CCM contained approximately twice as much hyaluronic acid, which was further discussed as a significant factor that enables the hNPCs to differentiate into chondrogenic and osteogenic lineages.

## 4. Discussion

The cumulus cells are so valuable that they directly affect the oocyte nuclear maturation, fertilization, and embryological development (Tahajjodi et al., 2019). Studies showed that the removal of cumulus cells caused failure in in vitro maturation (IVM) in various species including murine, bovine, and porcine (Wongsrikeao et al., 2005). During the IVF procedure, a sperm is injected into an oocyte’s cytoplasm by a fine glass needle by “partial” removing or stripping CCs to have a better view and pinpoint the egg (Evison et al., 2009). In this study, CCs were isolated and verified for appropriate characterization. The expression of genes specific to CCs was verified in isolated cells, and the morphology of the cells was confirmed with photographs from previous studies. However, very few of the studies (only involved in bovine but not human) include microscopic images of cumulus cells (Sadat Tahajjodi et al., 2019; Chermuła et al., 2020) and attempt to decipher the morphology of these cells (Gajda et al., 2007). When evaluated in this context, microscopic images of human CCs are quite special and precious and have been introduced to the world of science exclusively. When the morphology of the hCCs was monitored under the microscope, the cells had a completely different morphology by the end of the week upon seeding. Initially, cells possess a star-shaped appearance and later become like spindles through a phenomenon called senescence. One explanation for this is that during the release of a mature egg from the ovary, the CCs remain around the egg (Carrell et al., 1993) but wither away via menstrual cycle in case of no fertilization. Another explanation for the findings might be that hCCs undergo apoptosis (Moffatt et al., 2002) under various conditions including the aging of females or exposure of CCs to sperm. Oocytes prevent CCs’ apoptosis by secreting factors like BMP15 and BMP6 which show antiapoptotic characteristics (Hussein et al., 2005). Since CCs accelerate the aging of oocytes, the CCs are expected to have a limited life span (Miao et al., 2005). A supportive study claims that the CCs can stay in the cell culture environment for a limited time too. Their study involves the analyses of gene expressions and suggestions of candidate genes associated with death and aging in CCs (Chermuła et al., 2020). Their studies and ours share a similar cell culture condition to grow the CCs and the cells in our study survived no more than a week. Considering the inherent duration of menstrual cycle of a healthy woman and a fact that mature oocytes have a limited lifespan, we expect human CCs to live around 7 days.

The presence of angiogenic markers (Cumming et al., 2006) in hCCs was also detected in this study since stromal blood vessels play a crucial role during nutrients and oxygen transferring to primordial and primary follicles by passive diffusion (Da Broi et al., 2018). The most common in vitro model for angiogenesis is with HUVEC cells (Yukawa et al., 2018). Our study suggests that CCs may be an alternative model since the cells expressed *VEGF* highly and formed the vasculature like tubes in matrigel environment. Considering its role in wound healing process, angiogenesis is an important step (Rodrigues et al., 2019) and the CCs might have an impact on promoting wound healing, which was also demonstrated in this study.

CCs is the enriched by hyaluronic acid (HA), synthesized by HAS2, in their extracellular matrix and help them to expand for meiotic maturation and gain the developmental competence of the egg (Nevoral et al., 2015). Even the smallest amount of hyaluronic acid is very valuable because it is a naturally derived GAG with a wide range of usage areas (Dovedytis, Liu, and Bartlett 2020) and yet the optimization of in vitro HA production is very tedious and expensive (Boeriu et al., 2013). The yield of bacterial fermentation is characteristically low (0.1 g/g glucose) in addition to its hardship to obtain high weight-HA (HW-HA) economically from bovine vitreous humor and rooster combs (Saranraj 2013). Thus, obtaining HA from discarded cumulus tissue is powerful. Therefore, considering the presence of HA, which is synthesized by CCs under the effects of various factors, the amount of it obtained in this study is critical and worth further investigation. Moreover, many in vivo studies and in vitro experiments have demonstrated that HA fragments promoted angiogenesis and wound healing. Scientists explain the underlying mechanisms as the interaction between CD44 and HA oligosaccharides showing a promitotic effect that stimulate MMP2 and MMP9 production. Through this way, cell invasion via ECM increases to help vessel sprouting (Pardue et al., 2008). As a result, maximum efficiency is obtained from tube formation and scratch assays suggesting CCs as an alternative model for angiogenesis that may be caused by the angiogenic properties of hyaluronic acid.

In addition to its effects on wound healing and angiogenesis, HA has a critical role in differentiation of cells into other lineages, including osteocytes and chondrocytes (Jakobsen et al., 2010; Maharjan et al., 2011; Simpson et al., 2016; Amann et al., 2017; Cañibano-Hernández et al., 2019). In light of this, decreasing cartilaginous properties of hNPCs during intervertebral disc degeneration (IVD) was enhanced with cumulus cell-derived HA-containing differentiation media. The stemness characteristics of hNPCs were further investigated and it was revealed that the cells had the multilineage potential which confirmed the previous findings (Jia et al., 2017; Zhao et al., 2017). To our knowledge, this study is the first and only that investigates human CCs exclusively and shows their potential to be used as a differentiating agent and stimulator of the chondrogenic capacity of hNPCs which may be a value for the treatment of intervertebral disc degeneration.

In conclusion, our study focused on the human cumulus cells from differential perspectives. Within this study, the conditioned media harvested from the cumulus cells are enriched in HA and enhanced the chondrogenic potential of hNPCs and may be used as an alternative cell-related therapy to treat chondral-degenerative diseases.

The capacity of human cumulus cells in the treatment of chondro-degenerated nucleus pulposus cells was scrutinized. According to our results, the high hyaluronic acid content of cumulus cells was discovered using human primary culture samples derived from patients. Interestingly, we discovered a potent differentiation-inducing effect of hyaluronic acid on nucleus pulposus cells that indicated stemness characteristics. Furthermore, not only the chondroid property of human nucleus pulposus cells was stimulated when they were cultured in a conditioned medium of human cumulus cells but also their ability to differentiate into osteocytes was enhanced. Most strikingly, our study unveiled a remarkable proangiogenic and wound healing effect of hyaluronic acid-rich human cumulus cells. Groups cultured in cumulus cell-conditioned medium displayed a significant increase both in the number of branches in tube formation assays and wound closure area in scratch assays compared to those in control cells. Finally, the observation where cumulus cells were able to form the endothelial cell-like branches on matrigel underscores these cells as a valuable and novel model in addition to the widely used angiogenesis model of HUVEC cells. Altogether, it might be suggested that the potential of cumulus derived molecules on many aspects such as differentiation, angiogenesis, and wound healing might be derived from the stem cell characteristics of cumulus cells (Nie et al., 2011).

Further studies might be involved in trying of these cellular materials on animal models. The potential of these cells and their cellular ingredients on increasing the chondrogenicity of nucleus pulposi cells as well as enhancing wound healing capacity might be enlightened in in vivo models; therefore, these cells, once considered trash, can be a treasure now.

## Supplementary

Figure S1 The histogram results of stem cell markers in hNPCs by flow cytometry analysis demonstrate the mesenchymal but not the hematopoietic characteristics of hNPCs.

Figure S2 Cell viability assay shows the nontoxic effect of CCM dilution at 1:1 ratio in hNPCs.

Figure S3 Hyaluronic acid quantification, the comparison of HA amount (ng/mL) between the experimental group (EG; CCM) and control groups, * p < 0.05.

## Figures and Tables

**Table 1:** Pimers for the characterization of CCs

Name of related gene	Primer sequence		Ref no.
Connexin 43 (CX43)	F	5′-TTCCTCTCTCGCCCCAC-3′	(Ciuffreda et al., 2016)
	R	5′-GGCCTAGAAAGCTTACCTT-3′	
Cyclooxygenase 2 (COX2)	F	5′TTCAAATGAGATTGTGGGAAAAT-3′	(Kojima et al., 2000)
	R	5′AGATCATCTCTGCCTGAGTATCTT-3′	
Hyaluronic acid synthase 2 (HAS2)	F	5′-ATCCCATGGTTGGAGGTGTT-3′	(Cai, Li, and Na 2011)
	R	5′-TGCCTGTCATCACCAAAGCT-3′	
Pentraxin (PTX3)	F	5′-CATCCAGTGAGACCAATGAG-3′	(Takagi et al., 2020)
	R	5′-GTAGCCGCCAGTTCACCATT-3′	
Vascular endothelial growth factor (VEGF)	F	5′-TTGCCTTGCTGCTCTACCTC-3′	(Xu et al., 2015)
	R	5′-AGCTGCGCTGATAGACATCC-3′	
Vascular endothelial growth factor receptor 1 (VEGFR1)	F	5′-CAGGCCCAGTTTCTGCCATT-3′	(Urbantat et al., 2021)
	R	5′-TTCCAGCTCAGCGTGGTCGTA-3′	
Vascular endothelial growth factor receptor 2 (VEGFR2)	F	5′-CCAGCAAAAGCAGGGAGTCTGT-3′	(Chung et al., 2004)
	R	5′-TGTCTGTGTCATCGGAGTGATATCC-3′	
Von Willebrand factor (VWF)	F	5′-CGGCTTGCACCATTCAGCTA-3	(Katarivas Levy et al., 2019)
	R	5′-TGCAGAAGTGAGTATCACAGCCATC-3′	
COL2A1	F	5′-ACCCCAATCCAGCAAACGTT-3	(Doroski, Levenston, and Temenoff 2010)
	R	5′-ATCTGGACGTTGGCAGTGTTG-3′	
OPN	F	5′-GCCGACCAAGGAAAACTCACT-3′	(Selvamurugan et al., 2017)
	R	5′-CTTACTTGGAAGGGTCTGTGGG-3′	
18S	F	5′-GTAACCCGTTGAACCCCATT-3′	(Nguyen et al., 2019)
	R	5′-CCATCCAATCGGTAGTAGCG-3′	

**Table 2:** Gene expression data of 5 different samples (S: Sample): Genes specific to cumulus cells were depicted.

Genes	S1	S2	S3	S4	S5
*COX2*	+	+	+	+	+
*HAS2*	+	+	+	+	+
*PTX3*	+	+	+	+	+
*CX43*	+	+	+	+	+
*VEGF*	+	+	+	+	+
*VEGF-R1*	+	+	+	+	+
*VEGF-R2*	+	+	+	+	+
*VWF*	+	+	+	+	+
*18S*	+	+	+	+	+

**Table 3:** Flow cytometry analysis of hematopoietic surface markers for hNPCs. Flow cytometry analysis of mesenchymal surface markers for hNPCs

Hematopoietic markers	
Marker name	Gated % (±std)
CD31	1.04 (±0.1249)
CD14	1.09 (±0.24)
CD34	1.37 (±0.14)
CD45	1.16 (±0.37)
CD117	1.39 (±0.07)
**Mesenchymal markers**	
**Marker name**	**Gated % ( ±std)**
CD29	99.74 (±0.30)
CD44	99.96 (±0.045)
CD73	98.34 (±2.12)
CD90	96.92 (±1.52)
CD105	18.68 (±2.4)
