# Supplementary figures and images for: The investigation of diverse physiological and therapeutic impact of cellular-based products derived from human cumulus cells
Source: Turk J Biol. 2022 Sep 19;46(5):400–13. doi: 10.55730/1300-0152.2626 (PMC10388000; doi:10.55730/1300-0152.2626)

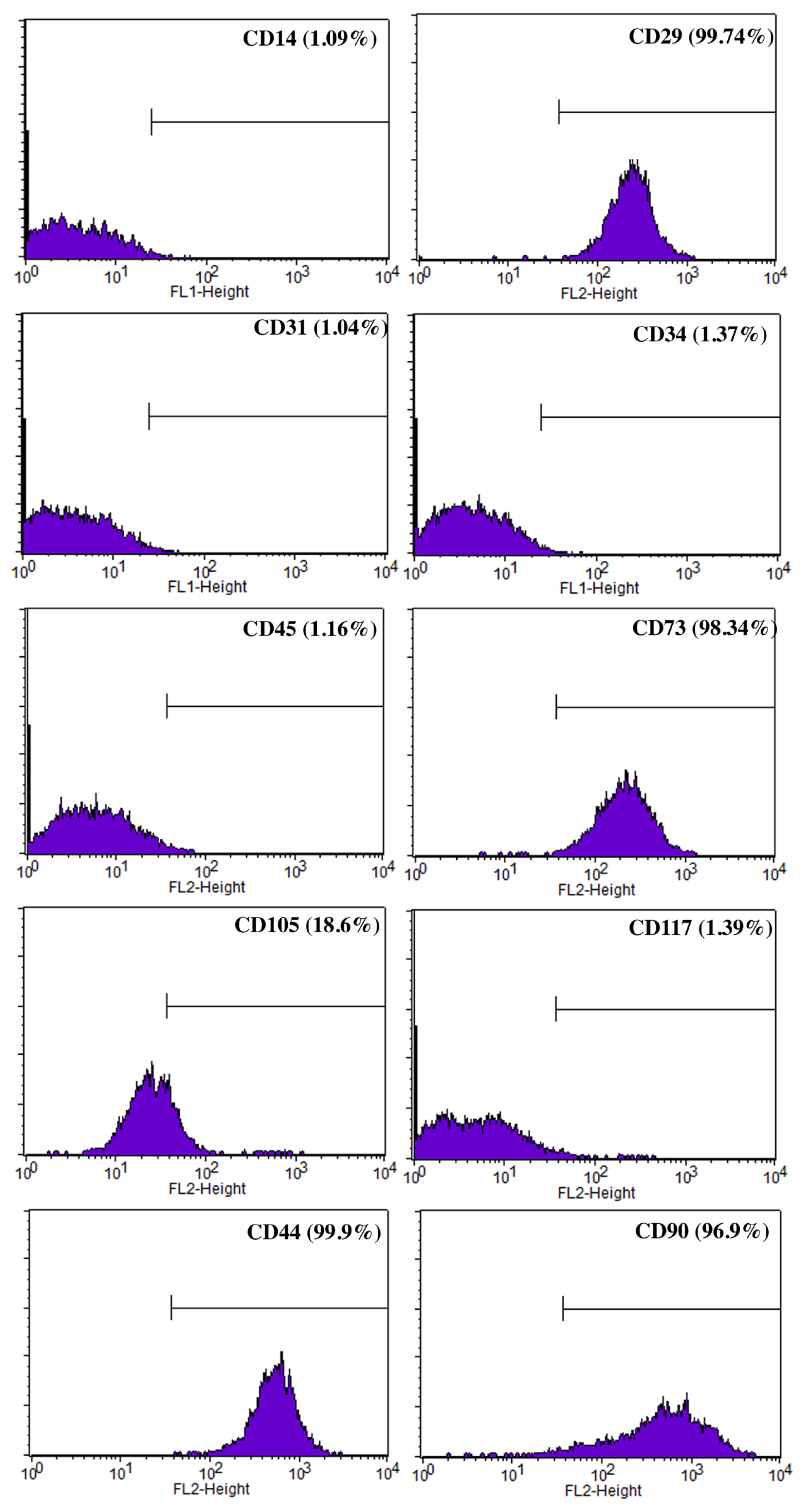

Supplement: Figure S1 — The histogram results of stem cell markers in hNPCs by flow cytometry analysis demonstrate the mesenchymal but not the hematopoietic characteristics of hNPCs. [file turkjbiol-46-5-400s1.tif]

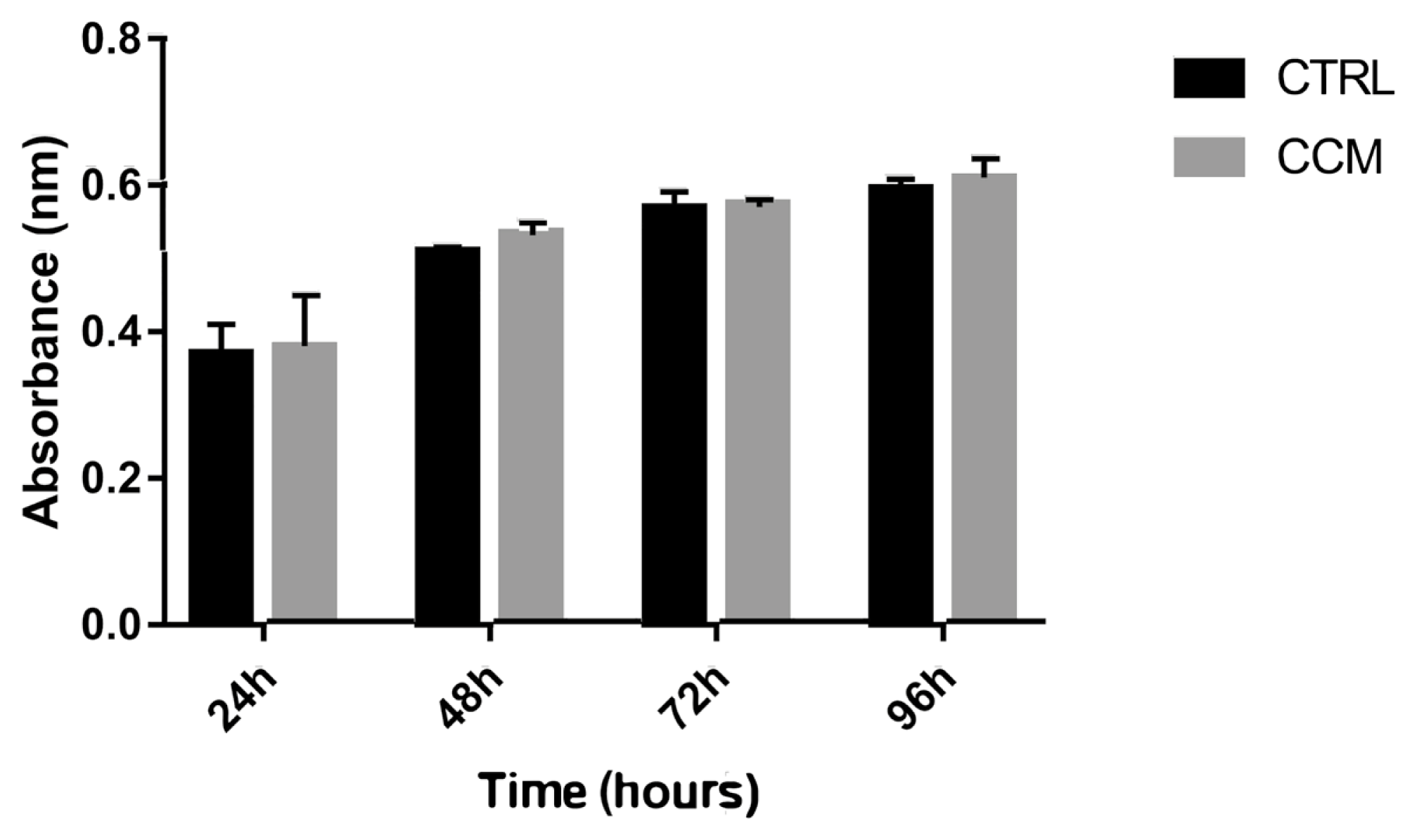

Supplement: Figure S2 — Cell viability assay shows the nontoxic effect of CCM dilution at 1:1 ratio in hNPCs. [file turkjbiol-46-5-400s2.tif]

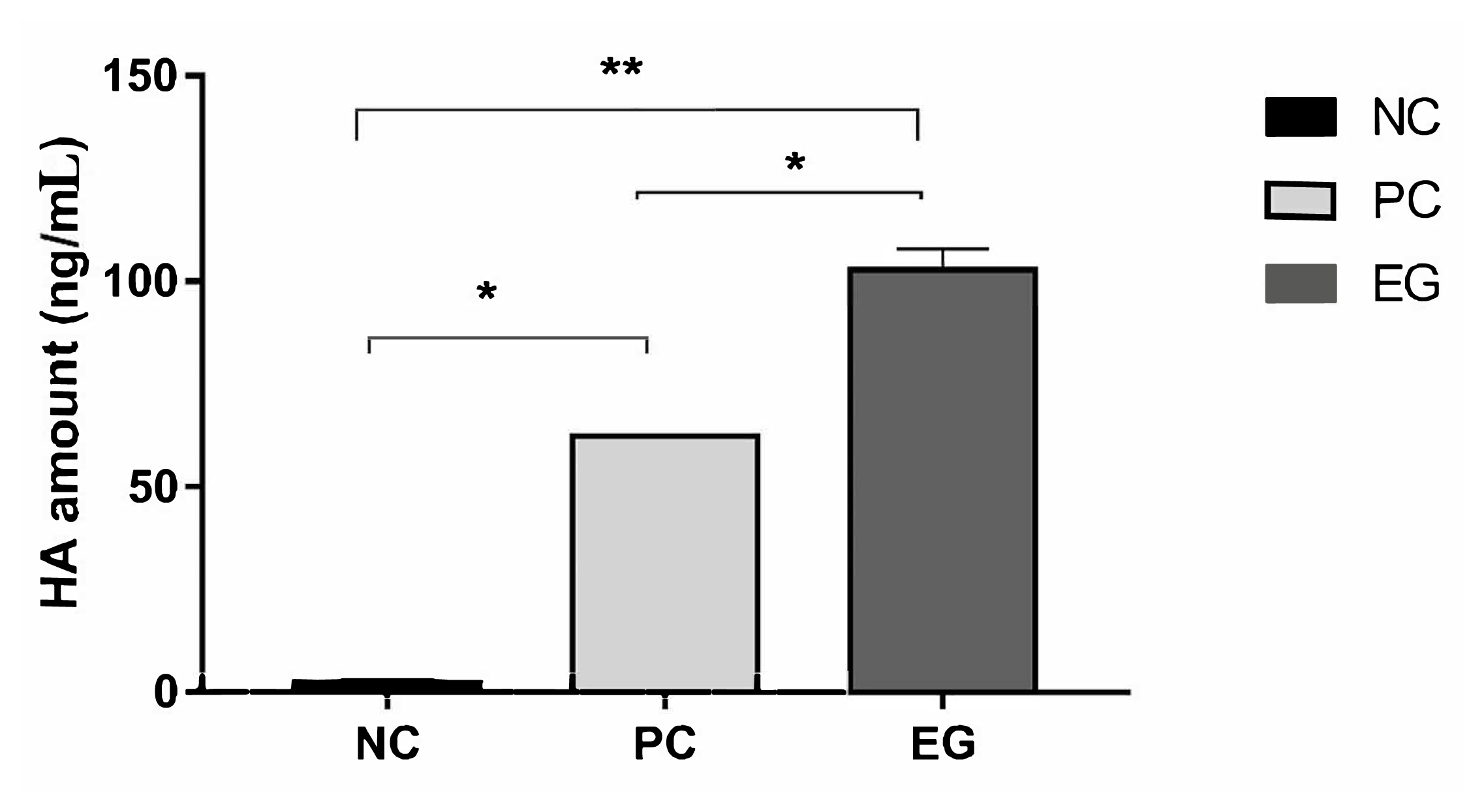

Supplement: Figure S3 — Hyaluronic acid quantification, the comparison of HA amount (ng/mL) between the experimental group (EG; CCM) and control groups, * p < 0.05. [file turkjbiol-46-5-400s3.tif]
